# A novel subtype of primary prostatic adenocarcinoma: A case report

**DOI:** 10.3892/ol.2013.1557

**Published:** 2013-09-02

**Authors:** XIAOQING YANG, CHEN XU, JIANING GUO, CHUNRUI YANG, YUMING YANG, RUIFA HAN

**Affiliations:** 1 Tianjin Institute of Urology, Department of Urology, Second Hospital of Tianjin Medical University, Hexi, Tianjin, P.R. China; 2 Department of Endocrinology, Second Hospital of Tianjin Medical University, Hexi, Tianjin, P.R. China; 3 Department of Colorectal Surgery, Affiliated Hospital of Nankai University, Hongqiao, Tianjin, P.R. China

**Keywords:** adenocarcinoma of the prostate, histopathological feature, urothelial carcinoma

## Abstract

The present study reports the novel case of an 81-year-old male with prostatic adenocarcinoma (PAC), whose histopathological study revealed a pure urothelial carcinoma (UC) that originated, however, from the prostatic glandular epithelium. The levels of serum prostate-specific antigen (PSA) were extraordinarily high in this patient. An MRI scan indicated a prostatic neoplasm and no malignant changes were observed in the bladder or other areas of the urinary tract. Hematoxylin and eosin-stained sections revealed a diagnosis of pure UC with no other form of differentiation (typical adenocarcinoma or squamous differentiation). The immunohistochemical findings were positive for PSA and P504S, and negative for CK7, CK20, 34βE12 and p63. A diagnosis of primary PAC (solid carcinoma) originating from the prostate was made based on the clinical, histopathological and immunohistochemical observations. This case was susceptible to diagnostic errors, however, a novel subtype of primary PAC was identified and termed the UC-like subtype.

## Introduction

Due to the anatomical proximity, urothelial carcinoma (UC) of the bladder may infiltrate and involve the prostate. High-grade urothelial and prostatic carcinomas have overlapping morphological characteristics and clinical manifestations. Therefore, differentiating between the diagnoses of carcinoma of the urothelium and adenocarcinoma of the prostate may be difficult. Usually, adenocarcinoma may be distinguished from transitional cell carcinoma exclusively by its histological aspect. However, if the histopathological features of the adenocarcinoma of the prostate are similar to those of UC, the differential diagnosis is difficult (1). The present study reported a novel subtype of prostatic adenocarcinoma (PAC) with rare histopathological features of pure UC. To the best of our knowledge, this is the first study to report PAC with rare histopathological features of pure UC. Written informed consent was obtained from the patient.

## Case report

**Figure 1:**
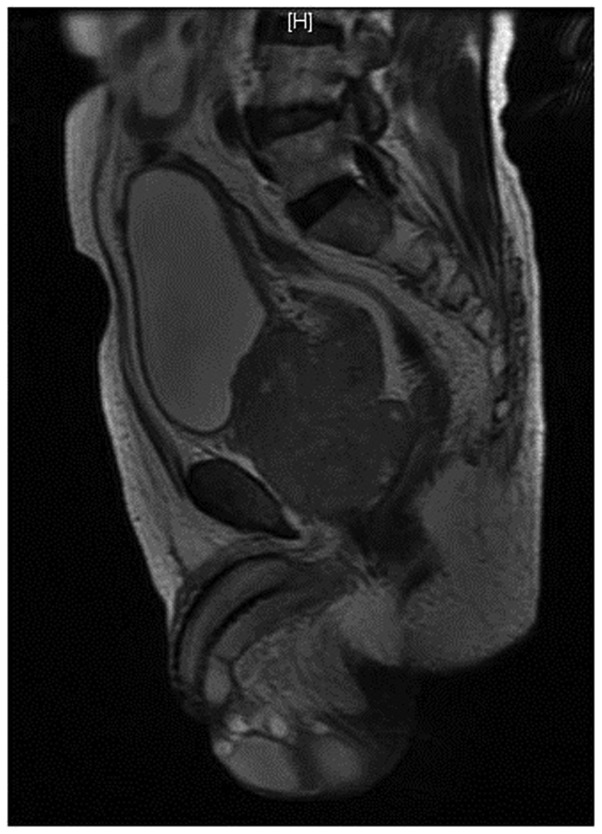
MRI scan showing the prostatic neoplasm, without a neoplasm of the bladder or other areas of the urethra, but with skeletal metastasis.

**Figure 2:**
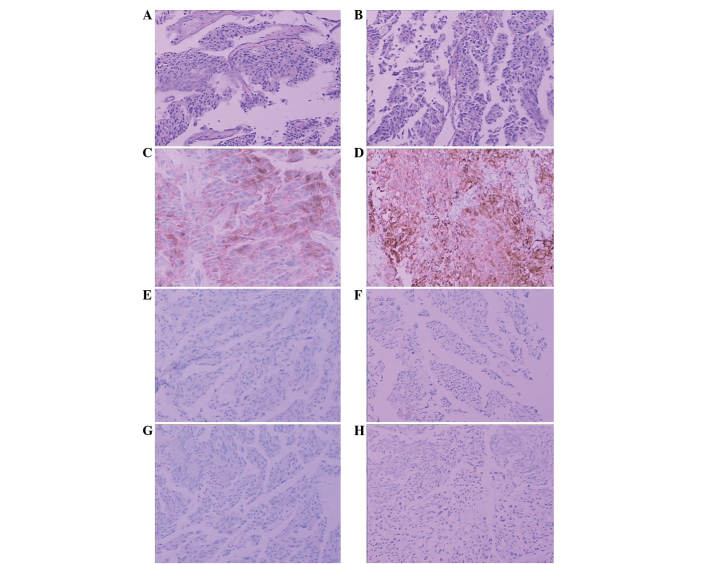
(A and B) UC-like structures with tumor cells that are lamellar in growth. Gradually maturing pattern of differentiation from the bottom to the surface, reflecting the hierarchical structure of the transitional epithelium. The nuclei display a polarity (HE). Scattered cancer cells strongly positive for (C) PSA and (D) P504s, and negative for (E) 34βE12, (F) CK7, (G) CK20 and (H) p63. UC, urothelial carcinoma; HE, hematoxylin and eosin; PSA, prostate-specific antigen; CK, cytokeratin. Magnification (C and D), ×200; (A, B, E, F and G), ×100.

An 81-year-old male presented to the Urological Outpatient Department with complaints of a recent onset of gross hematuria and obstructive symptoms. The patient denied smoking and the consumption of alcohol. The past medical history included a myocardial infarction and psoriasis. Subsequently, an enlarged prostate gland, indicative of a neoplasm, was identified using urological ultrasound. A transrectal ultrasound revealed an irregular prostatic enlargement of 6.6×6.4×7.6 cm. The serum prostate-specific antigen (PSA) and free PSA levels were >120 ng/ml (normal range, <4.0 ng/ml). An MRI scan (Fig. 1) revealed a prostatic neoplasm and pelvic lymph node enlargement, with invasion of the seminal vesicle. There was no neoplasm of the bladder or other areas of the urethra, but the patient was positive for skeletal metastases. A transrectal ultrasound-guided prostatic biopsy was executed and the specimens were submitted for histopathological evaluation. The pathological examination revealed a pure UC (Fig. 2) with the presence of solid nests of cells associated with dense or abundant cytoplasm and striking nuclear pleomorphism, and the absence or rarity of glandular lumina. No other differentiation (typical adenocarcinoma or squamous differentiation) was observed. In order to define the origin of the tumor cells and reach a diagnosis, immunohistochemical analyses were performed for PSA, high molecular weight cytokeratin (HMWCK; clone 34βE12), α-methylacyl coenzyme A racemase (AMACR/P504S), cytokeratin (CK)-7, CK 20 and p63 (Fig. 2).

The tissue specimens were fixed in 10% buffered formalin and the paraffin sections were stained with hematoxylin and eosin (HE) for conventional histology. The immunohistochemical staining was performed on representative sections of 5 μm in thickness using the labeled streptavidin biotin technique for the following antibodies: PSA (1:100 dilution; ZSGB-BIO, Beijing, China); α-methylacyl coenzyme A racemase (1:80 dilution; ZSGB-BIO); CK7 (1:50 dilution; ZSGB-BIO), CK20 (1:50 dilution; ZSGB-BIO); and HMWCK (34βE12; 1:100 dilution; DAKO, Carpinteria, CA, USA). Antibody detection was performed using the DAKO EnVision-System, with 3,3′-diaminobenzidine as a chromogen. Adequate positive and negative tissue controls were used throughout. The intensity of the immunohistochemical staining was evaluated semi-quantitatively using the following system: +++, strong, diffuse staining; ++, moderate staining; +, weak and focal staining; and −, no staining.

The histopathological analysis revealed UC-like structures with tumor cells that were lamellar in growth. Gradually maturing patterns of differentiation were observed from the bottom to the surface of the sections, which reflected the hierarchical structure of the transitional epithelium. The nuclei displayed a polarity, as observed by the HE staining. The immunohistochemical findings are summarized in Table I. The carcinoma component was positive for PSA and P504S, but negative for 34βE12, CK7, CK20 and p63 (Fig. 2). A diagnosis of a primary PAC (solid carcinoma), Gleason 8 grade (4A + 4A) with a prostatic origin, was made based on the clinical features, serum PSA level and immunohistochemical findings. The patient was treated with maximal androgen blockade, including luteinizing hormone releasing-hormone analogue (3.65 mg/28 days) and bicalutamide (50 mg qd). At three years post-diagnosis, the patient underwent a transurethral resection of the prostate (TURP) to relieve the obstructive symptoms. A pathological examination of the resected specimens revealed UC, and the immunohistochemistry of PSA, 34βE12, P504S, CK7, CK20 and p63 also yielded the same results.

## Discussion

Since prostate cancer or UC may occur in elderly men synchronously or metachronously, the differential diagnosis of a poorly-differentiated carcinoma of the bladder or prostate includes urothelial and prostate carcinoma (2). An accurate distinction between the two has significant therapeutic and prognostic ramifications. A PAC may respond to hormonal therapy and should not be treated by a cystoprostatectomy. An appropriate diagnosis also determines the stage for prognostication. In problematic cases, immunohistochemistry may be used to aid in the identification of the origin of the tumor cell when the morphological features on HE stained sections are unclear. The present study analyzed patients with UC and primary transitional cell carcinoma (TCC) components in prostatic tissue sections using studies obtained from the literature (Table II). The tumors were primary PACs with coexisting UC, which originated from the urothelium. PAC or pure UC/TCC were shown on HE sections. The present case varied from those cases that were reported in the literature, as it was pure UC, as shown by HE sections, originating in the prostatic glandular epithelium. UC is distinguished from poorly-differentiated PAC by its histopathological characteristics, including the presence of solid nests of cells associated with dense or abundant cytoplasm and striking nuclear pleomorphism, with the absence or rarity of glandular lumina. The serum free PSA level is a main marker for prostate cancer screening. However, in the present case, the serum PSA level was high (1,130 ng/ml), indicating prostate cancer. UC was detected by transurethral biopsy. A bone ECT revealed multiple bone metastases. Due to these factors, achieving a definite diagnosis was difficult. In order to identify the origin and character of the prostatic cancer, a panel of immunohistochemistry was selected.

PSA is a 33-kDa serine protease that is secreted by the prostatic epithelium. The sensitivity and specificity of PSA is high in prostate cancer, at 100% sensitivity. In poorly-differentiated prostate cancer and PAC, the expression levels of PSA may reach 85–95%. PSA is the oldest and most commonly used immunohistochemical marker to identify cancers of prostatic origin (3). The basal cell marker, 34βE12, is a protein cloned by HMWCK, and is useful for the observation of basal cells, as their presence contradicts a diagnosis of prostatic carcinoma (4).

p63 is expressed in the majority of UCs, but is not present in the majority of PACs. The protein may be used as a reliable marker to distinguish PACs from UCs in difficult cases in conjunction with other markers, such as PSA (5). In order to obtain an optimal immunohistochemical panel to distinguish poorly-differentiated PAC from UC, Kunju *et al*(1) analyzed a panel consisting of PSA, prostatic acid phosphatase (PAP), 34βE12, CK7, CK20, p63 and P504S. The results indicated that PSA stained 95% of prostate cancer vs. 0% of UC cases. 34βE12 and p63 stained 97% and 92% of UC vs. 2% and 0% of PAC cases, respectively. A panel of PSA, 34βE12 and p63 was optimal for separating 95% PAC (PSA^+^/34βE12 and/or p63^−^) vs. 97% UC (PSA^−^/34βE12 and/or p63^+^) (1). P504S is a metabolic enzyme whose overexpression has been shown to be a diagnostic indicator of PAC and other solid tumors. The prostate and basal cell biomarker, P504S, has been used together with the morphology to assist in the formation of a diagnosis in diagnostically suspicious cases, with a very high sensitivity and specificity. This has increased the diagnostic accuracy of prostate cancer worldwide. A binding of 34βE12 and P504S is of great value in diagnosing morphologically suspicious cases and significantly increasing the 34% diagnostic accuracy in prostate cancer (14). In the present case, basal cell staining recorded positive results for PSA and P504S, supporting the definitive diagnosis of malignant PAC. 34βE12 and p63 were negative in this case, discouraging a diagnosis of UC.

CK7 and CK20 are also useful markers to distinguish PAC from UC. Bassily *et al* studied the expression of CK7 and CK20 in PAC and UC, and estimated their usefulness for distinguishing between the two tumors. In the prostatic and metastatic tumors, neither were positive for the markers. However, 61% of the UC cases were positive for CK7 and CK20 (15). The present case was also negative for CK7 and CK20.

The common subtype of PAC is acinar adenocarcinoma, however, several rare subtypes exist, including atrophic, pseudohyperplastic variant, foamy, colloid, signet-ring, oncocytic, lymphoepithelioma-like and sarcomatoid carcinoma. Due to the observations in this novel case of PAC, in which histopathological study revealed pure UC, with, however, a prostatic glandular epithelial origin, a new subtype of adenocarcinoma of the prostate, termed the UC-like subtype, was identified. The distinction between the UC-like subtype of PAC and UC is significant, since the latter is associated with multifocal lesions in the urinary tract and requires a alternative form of therapy.

## Figures and Tables

**Table I:** Immunohistochemical observations.

Antibody	PSA	P504S	34βE12	CK7	CK20	p63
Area of cancer	+++	+++	−	−	−	−

PSA, prostate-specific antigen; CK, cytokeratin; +++, strong, diffuse staining; −, no staining.

**Table II:** Patients with UC involving the prostate.

First author, year (ref.)	n	Age, years	Site	Histopathological feature	Immunohistochemistry of UC							Origin	Diagnosis

					PSA	PAP	CK7	CK20	34βE12	CEA	p63		
Hashimoto *et al,* 1989 (6)	1	58	Prostate	TCC	−							Prostate	TCC-P
Mottola *et al,* 1991 (7)	3	-	Prostate	TCC								Prostate	TCC-P
Mai *et al,* 2002 (8)	6	-	Prostate	UC and AC	−/+	+/++	−/+	−		−/+		Prostate/urinary	PAC
Morikawa *et al,* 2003 (9)	1	77	Prostate	TCC	−							Prostate	TCC-P
Ushida *et al,* 2004 (10)	1	77	Prostate	PAC and TCC	+/−								PDC
Huang *et al,* 2004 (11)	1	83	Bladder	PAC and UC	−	−	+	+	+			Prostate	PAC
Curtis *et al,* 2005 (12)	1	89	Prostate	AC and UC	−	−	++	++	+	++		Prostatic urethra	UTA
Martínez *et al,* 2007 (13)	1	-	Prostate	PAC and UC	−	−	++	++	+++		+++	Urinary bladder	PAC

UC, urothelial carcinoma; PSA, prostate-specific antigen; PAP, prostatic acid phosphatase; CK, cytokeratin; CEA, carcinoembryonic antigen; PAC, prostatic adenocarcinoma; TCC, transitional cell carcinoma; UTA, urothelial-type adenocarcinoma arising in the prostatic urethra or proximal prostatic ducts; TCC-P, primary transitional cell carcinoma of the prostate; PDC, prostatic duct carcinoma; −, no staining; + weak and focal staining; ++, moderate staining; +++; strong and diffuse staining.
